# The VirusBanker database uses a Java program to allow flexible searching through *Bunyaviridae *sequences

**DOI:** 10.1186/1471-2105-9-83

**Published:** 2008-02-05

**Authors:** Mathieu Fourment, Mark J Gibbs

**Affiliations:** 1 Department of Biological Sciences, Macquarie University, Sydney NSW 2109, Australia; 2 School of Botany and Zoology, The Australian National University, Canberra ACT 0200, Australia

## Abstract

**Background:**

Viruses of the *Bunyaviridae *have segmented negative-stranded RNA genomes and several of them cause significant disease. Many partial sequences have been obtained from the segments so that GenBank searches give complex results. Sequence databases usually use HTML pages to mediate remote sorting, but this approach can be limiting and may discourage a user from exploring a database.

**Results:**

The VirusBanker database contains *Bunyaviridae *sequences and alignments and is presented as two spreadsheets generated by a Java program that interacts with a MySQL database on a server. Sequences are displayed in rows and may be sorted using information that is displayed in columns and includes data relating to the segment, gene, protein, species, strain, sequence length, terminal sequence and date and country of isolation. *Bunyaviridae *sequences and alignments may be downloaded from the second spreadsheet with titles defined by the user from the columns, or viewed when passed directly to the sequence editor, Jalview.

**Conclusion:**

VirusBanker allows large datasets of aligned nucleotide and protein sequences from the *Bunyaviridae *to be compiled and winnowed rapidly using criteria that are formulated heuristically.

## Background

Virus sequence databases are known for their well-organized interfaces and search systems; important examples include the Influenza Virus Resource of GenBank [1], the Viral Bioinformatics Resource Centre of the Universities of Victoria and of Alabama at Birmingham [2], and the HIV, influenza and Hepatitis C databases of the Los Alamos National Laboratory [3]. These databases allow a user to use quite complex search criteria to define large datasets of sequences. In almost all of these databases records are represented on webpages and selections are made using standard webpage menus and check boxes. Sorting processes are done through calls to a database on a server. The use of web-pages, however, has some disadvantages: a user cannot build a dataset in successive steps, where some sequences are added and others are deleted, and check-boxes are slow to use. As a result a user may be discouraged from exploring the database and may fail to identify some sequences they wish to include or exclude, or they may choose not to use the database to fully define the dataset but instead use downstream editing software to check and refine it.

## Construction and content

### Systems and methods

**Figure 1:**
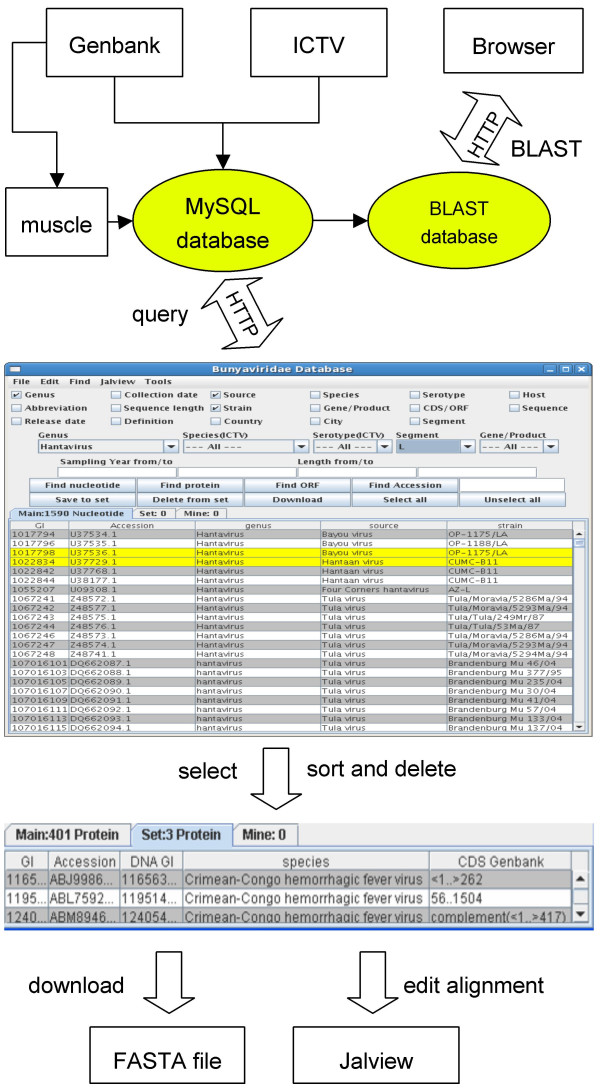
**VirusBanker Flowchart.** VirusBanker consists of a Java program interacting with a MySQL database through the internet. The database is updated and curated using information available on GenBank and the ICTV website. Sequences aligned using MUSCLE are linked to the sequence records. Once a dataset is selected sequences can be saved to a file or edited directly with Jalview. A blast server is accessible through a web browser.

In the VirusBanker database, an alternative programmatic approach has been used. VirusBanker consists of a Java client-server program (Figure 1) that generates an interface consisting of three spreadsheets. The sequence characteristics required for sorting are loaded from a MySQL database on a server when the program is launched. The program does not call the server during a sorting operation, but only when a set is selected and saved to the second spread sheet, or downloaded, or the user changes between viewing protein and nucleic acid sequence data. Sequences are represented as rows that may be scrolled through. A user can choose from a menu of 16 characteristics that once selected are represented as columns. Sequence records are sorted alphabetically or numerically by clicking the column titles. Sets of sequences are selected by clicking and dragging the cursor, and saving the selected sequences to a second spreadsheet. A click on the Geninfo Identifier number (GI) opens the record from GenBank in a web browser. A series of drop-down menus may also be used to define a sequence set or search for a particular sequence by accession code or GI number.

Once the user has saved a selection of sequences to the second spreadsheet, the same range of selection and sorting operations are available. Individual sequences or sets of sequences may be moved to the second spreadsheet or deleted from it, without a call to the database. The aim of the approach is to permit a user to rapidly explore the database, and create the selection criteria while learning about the features of the virus sequences and so better refine the dataset. A third spreadsheet is available to display sequences uploaded by the user, which is useful, for example, to review the related GenBank records or to identify the protein sequences encoded by nucleotide sequences. These operations are possible after the user has manually cross matched the GI numbers of the uploaded sequences with the GI numbers held in VirusBanker. The Java program and database software have been tested with a dataset of more than 40,000 sequences: no difficulties were experienced except for slight delays in sorting processes.

The VirusBanker program directly connects with the open source sequence editor Jalview [4]. Sequences and alignments may be passed from the second spreadsheet to the editor, so that Jalview's phylogenetic, clustering and alignment functions are immediately accessible. Sequence sets may also be downloaded in FASTA or NBRF/PIR formats. Nucleotide sequences that match the coding sequences in GenBank (cds and ORFs) are identified automatically and are also available. VirusBanker provides a link to a web page for querying sequences using the Basic Local Alignment Search Tool (BLAST) against precompiled nucleotide and protein databases [5]. This may be used to help identify newly sequenced virus isolates or to identify closely related sequences within the database.

### Database content

The *Bunyaviridae *consists of five genera: *Orthobunyavirus*, *Nairovirus*, *Phlebovirus*, *Tospovirus *and *Hantavirus*. The viruses have enveloped virions and single-stranded, negative-sense RNA genomes that consist of small (S), medium (M) and large (L) segments, comprising from 11000 to 28000 bases of sequence in total, and encoding four to six proteins. Viruses from the family that cause significant disease include Rift Valley fever virus (RVFV) [6] that spreads in people and livestock in Africa and the Arabian Peninsula, Crimean-Congo haemorrhagic fever virus (CCHFV) [7] that spreads in Africa, eastern Europe and Asia, and Hantavirus [8,9], that spreads in the Americas, eastern Europe, and eastern Asia. Fatality rates as high as 50% may be suffered during outbreaks of disease caused by RVFV and CCHFV. One unusual aspect of the family is that it includes a genus of plant viruses: *Tospovirus*. Tomato spotted wilt tospovirus (TSWV), the best known of these plant viruses, is a significant crop pathogen [10]. Most viruses from the family also infect arthropods that act as vectors. Most of the vertebrate-infecting members of the family are transmitted by mosquitoes, ticks, or phlebotomine flies. Tospoviruses are transmitted by thrips. Hantaviruses are mainly transmitted in aerosols. Transovarial and venereal transmission has been demonstrated for some species.

The characteristics used in the *Bunyaviridae *VirusBanker database are those often used in studies of virus sequences and include the genus, species, strain, host and country names, collection dates, and gene and protein names. Protein sequences were those identified in GenBank and protein sequence names were curated for consistency. A customized pipeline consisting of a set of Java interfaces was used to create, curate and update the database. The original data was retrieved from GenBank using the ENTREZ Programming Utilities (eUtils), then it was parsed with a Java program and manually curated. Species names were obtained from the most recent publication of the International Committee on Taxonomy of Viruses [11] using Perl scripts. The database program has been running for 3 years and has been used by biologists and bioinformaticians. The *Bunyaviridae *database is updated every 6 months or upon a user's request and is the program is frequently updated.

Proteins and nucleotide alignments used in the *Bunyaviridae *VirusBanker database were generated using MUSCLE [12]. The first and last 10 residues of the sequences and sequence lengths are also provided as characters, which is useful since the *Orthobunyavirus*, *Nairovirus*, *Phlebovirus*, *Tospovirus *and *Hantavirus *have different conserved terminal sequences [11], and although the majority of the nucleotide and protein sequences in the database are incomplete many them have matching terminal subsequences. The source and definition fields from GenBank are recorded as separate columns in VirusBanker to provide further taxonomic information. More information on hosts and vectors is available from another database [13] and this should be used in the future, but at present too few sequence records are linked to such data.

## Utility and discussion

The routine use of reverse transcription PCR and sequencing to detect and characterise RNA viruses has led to a dramatic increase in the number of full and partial genomic sequences submitted to the primary public databases. However, building coherent and complete datasets from the public databases has become increasingly difficult because of the large number of sequences and the lack of consistency in their annotation. Researchers sometimes use incorrect or unofficial species names and different names for homologous genes and proteins. Very useful databases have been created for well known viruses such as HIV and influenza, but less attention has been given to the *Bunyaviridae *and other virus groups. The Viral Orthologous Cluster database, which was based on the Poxivirus Orthologous Clusters database [14], also uses a Java client-server application and contains data from the *Bunyaviridae *and it appears to be designed for analysing complete genomes. The Virus Orthologous Cluster database is limited to 981 full segment sequences, it does not offer aligned sequences, but rather offers to align selected sequences, and it does not provide easy access to the terminal sequences nor contain information on the host and location of isolation.

## Conclusion

We have created a database containing 3958 nucleotide sequences and 4267 protein sequence from the *Bunyaviridae *that were released in GenBank before September 2007. By providing a spreadsheet of the data that is available in GenBank and from the ICTV, VirusBanker allows users to quickly define large and coherent datasets with fairly complex criteria and in this way it may speed up the analysis of these important viruses. Some operations are faster with VirusBanker than with equivalent HTML pages: including or excluding sequences from a subset, reversing such an operation, ordering and reordering sequences, and switching between spreadsheets. The speed with which these operations can be done encourages the user to refine datasets using VirusBanker rather than some downstream editing software.

## Availability and requirements

VirusBanker is freely available at  and . Java JRE 1.4 or above is required.

## Authors' contributions

MF implemented the program and the database. MF and MJG planned the project and drafted the manuscript. Both authors read and approved the final manuscript.
